# Real-world efficacy and safety of dolutegravir plus lamivudine versus tenofovir plus lamivudine and efavirenz in ART-naïve HIV-1-infected adults

**DOI:** 10.1097/MD.0000000000031100

**Published:** 2022-10-21

**Authors:** Jin Li, Dabiao Chen, Zhiwei Wen, Yanzhang Du, Zhanlian Huang, Huijun Zhong, Yanhao Wang, Sichun Yin

**Affiliations:** a Department of Infectious Diseases, The Ninth People’s Hospital of Dongguan, Dongguan, China; b Department of Infectious Diseases, The Third Affiliated Hospital of Sun Yat-sen University, Guangzhou, China.

**Keywords:** ART-naïve, dolutegravir, DTG + 3TC, dual therapy, HIV-1 DNA, lamivudine

## Abstract

Limited real-world data on dolutegravir (DTG) plus lamivudine (3TC) for HIV-1-infected individuals have been reported. This study aimed to evaluated the real-world efficacy and safety of DTG + 3TC in ART-naïve HIV-1-infected adults in China. This real-world prospective observational cohort study enrolled HIV-1-infected adults receiving ART initiation with DTG + 3TC (D3 group) or tenofovir plus lamivudine and efavirenz (TDF + 3TC + EFV, TLE group) with subgroups of low viral load (LVL, ≤500,000 copies/mL) and high viral load (HVL, >500,000 copies/mL) according to baseline HIV-1 RNA. Efficacy were assessed by proportion of virologic suppression, changes of CD4^+^ cell count and CD4/CD8 ratio, HIV-1 DNA decay, and safety by symptoms and changes of laboratory indicators at week 4, 12, 24, 36, and 48. Totally 45 participants in D3 group and 95 in TLE group were enrolled. The proportion of HIV RNA < 50 copies/mL were 48.7% (19/39), 84.6% (33/39), 100% (39/39), 100% (39/39) in D3-LVL subgroup at week 4, 12, 24, 48, compared with 1.3% (1/75), 14.7% (11/75), 86.7% (65/75), 96.0% (72/75) in TLE-LVL subgroup, with *P* < .05 at week 4, 12, and 36. The proportion were 0.0% (0/6), 66.7% (4/6), 83.3% (5/6), 100% (6/6) in D3-HVL subgroup compared with 0.0% (0/20), 5.0% (1/20), 85.0% (17/20), 100% (20/20) in TLE-HVL subgroup, with *P* < .05 at week 12. No virologic rebound was observed in D3 group. Mean change of CD4/CD8 ratio were higher in D3-LVL versus TLE-LVL subgroup at each scheduled visit (*P* < .05), while CD4^+^ cell counts increased significantly in D3-HVL versus TLE-HVL subgroup at week 4 and 12 (*P* < .05). Less complaint of dizziness, insomnia, dreaminess and amnesia, lower elevated level of triglyceride and higher elevated level of creatinine from baseline to week 48 were documented in D3 group (*P* < .05). Total HIV-1 DNA decayed along with HIV-1 RNA after DTG + 3TC initiation in both D3-LVL and D3-HVL subgroups. DTG + 3TC achieved virological suppression more rapidly and stably versus TDF + 3TC + EFV in ART-naïve HIV-1-infected adults, with better immunological response and less adverse drug effect, and reduced total HIV-1 DNA effectively. DTG + 3TC is a potent regimen for ART-naïve individuals with HIV-1 infection.

## 1. Introduction

Human immunodeficiency virus (HIV) infection has become a manageable chronic condition since the advent of combination antiretroviral therapy (ART). However, it remains a major public health crisis worldwide. There were approximately 37.7 million people living with HIV at the end of 2020 with 1.5 million people newly infected in 2020 globally.^[1]^ In China, more than 1 million people were reported to live with HIV-1 by 2020, the majority of whom were receiving the combination of tenofovir (TDF), lamivudine (3TC) and efavirenz (EFV) (TDF + 3TC + EFV) as first-line free regimen to obtain virological response and reduce mortality.^[2]^ However, quite a few of them suffer from long-term side effects such as dizziness, sleep disorder, etc., higher risk of drug-drug interactions or problems of drug resistance and virologic failure.

Dolutegravir (DTG) plus lamivudine (DTG + 3TC) has been recommended as another first-line treatment in recent guidelines^[2–5]^ due to its non-inferiority antiviral efficacy with high genetic barrier to resistance and lower potential for adverse drug effect in previous designed studies.^[6–10]^ But limited real-world data of DTG + 3TC therapy especially in China have been reported. Furthermore, DTG + 3TC and TDF + 3TC + EFV are not uncommonly found in individuals with HIV-RNA > 500,000 copies/mL, though neither of them was preferred in the guidelines. More clinical evidence should be accumulated and analyzed to verify their effectiveness.

Despite the success of ART, so far HIV cure cannot be achieved due to the persistence of HIV reservoir. The quantification of total HIV DNA is currently the most widely used marker for exploring the HIV reservoir.^[11,12]^ It was reported in previous studies that HIV-DNA decreases rapidly during the first year after ART initiation of some regimens not containing DTG, but afterwards the decay rate become slow and the HIV-DNA level remains almost stable for a long-time in virally suppressed patients.^[13,14]^ Considering that DTG has been proved for its effectiveness of inhibiting HIV RNA, DTG + 3TC could possibly be a potent ART regimen for HIV DNA suppression. However, to date, few data about HIV DNA response on DTG + 3TC have been found.

Thus, we conducted a prospective observational cohort study to evaluate the real-world efficacy and safety of DTG + 3TC compared with TDF + 3TC + EFV for treatment of HIV-1 infection in ART-naïve adults in China. Also, we monitored the dynamics of plasma HIV-1 DNA after DTG + 3TC initiation to explore its regularity.

## 2. Methods

### 2.1. Study design and participants

This ongoing real-world prospective observational cohort study enrolled individuals with HIV-1 infection who received initial ART regimens of either DTG + 3TC (D3) or TDF + 3TC + EFV (TLE) in the Ninth People’s Hospital of Dongguan from January 2020 to January 2021. Data were collected from January 2020 to January 2022.

Exclusion criteria included preexisting major viral resistance mutations to nucleoside reverse transcriptase inhibitors (NRTIs), non-nucleoside reverse transcriptase inhibitors, or integrase strand transfer inhibitors (INSTIs); coinfection with hepatitis B; and age ≥ 18 years.

### 2.2. Procedures and outcomes

The ART regimens were determined according to Chinese guidelines for diagnosis and treatment of HIV/Acquired Immune Deficiency Syndrome (2018 edition)^[15]^ and patients’ requirement.

Demographic and clinical data including age, gender, route of infection, opportunistic infections, comorbidity, combined drugs, weight, body mass index, HIV-1 RNA, CD4^+^ T lymphocyte count, CD4/CD8 ratio, blood routine, urine routine, liver function, renal function, fasting plasma glucose (FPG) and lipids were collected at baseline. Subgroups were divided according to baseline HIV-1 RNA, including low viral load (LVL, ≤500,000 copies/mL) and high viral load (HVL, >500,000 copies/mL). Genotypic tests were performed by the Chinese Center for Disease Control and Prevention prior to ART.

Study visits were scheduled at week 4, 8, 12, 24, 36, 48 following ART initiation. Symptoms, blood routine, urine routine, liver function, renal function, FPG, lipids, HIV-1 RNA and CD4^+^ T lymphocyte count were tested at each visit in 2 groups. HIV-1 DNA test was performed at baseline and week 4, 8, 12, 24, 48 in D3 group.

HIV RNA was extract from plasma using SUPBIO Nucleic Acid Extraction or Purification Kits. The HIV-1 RNA Quantitative PCR Kit (SUPBIO, Guangzhou, China) was used for quantitating HIV RNA. The linear quantification range of the HIV-1 RNA Quantitative PCR Kit was 12 to 6.0 × 10^6^ copies/mL. Total HIV-1 DNA was extracted from peripheral blood containing 0.25 to 1 million peripheral blood mononuclear cells (PBMCs) using the Qiagen QIAsymphony DNA Mini Kit (QIAGEN, Hilden, Germany). The SUPBIO total HIV-1DNA Quantitative PCR Kit (SUPBIO) was used for simultaneously quantitating total HIV-1 DNA and cell number. The linear quantification range of the SUPBIO total HIV-1 DNA quantitative kit was 20 to 1.0 × 10^5^ copies/10^6^PBMCs.

The primary outcome was the proportion of participants with virological suppression, defined as HIV-1 RNA < 50 copies/mL,^[3]^ at week 4,12, 24, 48. The secondary outcomes included change of CD4^+^ and CD4^+^/CD8^+^ as immunological response, change of alanine aminotransferase, aspartate aminotransferase, bilirubin, creatine, lipids and incidence of dizziness, insomnia and other symptoms as adverse drug reaction. Also, total HIV-1 DNA was tested for virological response analysis.

### 2.3. Statistical analysis

Continuous variables were presented as the mean ± SD or median (range) for descriptive analysis. Categorical variables were presented as percentages and frequencies. Differences were analyzed using t-test for continuous variables and chi-squared test or Fisher’s exact test for categorical variables. Correlation between HIV-1 DNA and HIV-1 RNA was assessed with Pearson correlation analysis. Statistical analyses were performed using the Statistical Package for the Social Sciences (SPSS) version 23.0 (IBM, Chicago, IL). All *P* values were 2 tailed, and *P* < .05 was considered to be statistically significant.

### 2.4. Ethical statement

The study protocol was approved by the Research Ethics Committee of Dongguan People’s Hospital (AF-97-04). All participants provided written informed consent prior to the initiation of study procedures. All study procedures were performed in accordance with relevant local guidelines and regulations.

## 3. Results

### 3.1. Baseline characteristics

Totally 140 ART-naïve HIV-infected participants were enrolled in this study from January 2020 to January 2021, including 45 in DTG + 3TC (D3) group and 95 in TDF + 3TC + EFV (TLE) group. No mutations conferring resistance to NRTIs, non-nucleoside reverse transcriptase inhibitors or INSTIs were detected prior to ART. Till January 2022, all participants have been regularly followed up for at least 48 weeks. No lost to follow-up visit or change in ART regimen was found.

Baseline demographic and clinical characteristics were generally similar between the 2 groups with the exception of age and proportions of comorbidity. Mean level of baseline HIV-1 RNA was lower in D3 group than in TLE group without statistical difference. In addition, no obvious difference was found between average baseline HIV-1 RNA in either LVL subgroups or HVL subgroups (Table 1).

**Table 1:** Comparison of baseline demographic and clinical characteristics in two groups.

	D3 (n = 45)	TLE (n = 95)	*P* value
Age (yr, mean ± SD)	33.8 ± 12.0	38.1 ± 11.9	.045*
Gender (male, %)	38 (84.4%)	83 (87.4%)	.637
Route of infection, n (%)			
Homosexual	33 (73.3%)	71 (81.0%)	.159
Heterosexual	9 (20.0%)	17 (17.9%)	
Others	3 (6.7%)	17 (1.1%)	
OIs, n (%)	0	0	*–*
Comorbidity, n (%)	8 (17.8%)	0	.000*
Combined drugs, n (%)	2 (4.4%)	0	.102
Weight (kg, mean ± SD)	60.7 ± 12.2	59.1 ± 8.0	.386
BMI (kg/m^2^, mean ± SD)	21.4 ± 3.4	21.1 ± 2.5	.518
HIV-1 RNA (×10^5^ copies/mL, mean ± SD)	1.75 ± 3.15	3.75 ± 5.74	.065
LVL, ≤500,000 copies/mL	39 (86.7%)	75 (78.9%)	.278
HVL, >500,000 copies/mL	6 (13.3%)	20 (21.1%)	.278
CD4^+^ count (cells/µL, mean ± SD)	342.2 ± 240.1	276.9 ± 185.8	.080
≤200	12 (26.7%)	33 (34.7%)	.439
>200	33 (73.3%)	62 (65.3%)	.439
CD4/CD8 ratio (mean ± SD)	0.29 ± 0.21	0.24 ± 0.17	.122
ALT (U/L, mean ± SD)	30.8 ± 30.7	25.2 ± 21.4	.218
AST (U/L, mean ± SD)	30.1 ± 32.1	25.7 ± 15.3	.270
TBIL (mmol/L, mean ± SD)	12.4 ± 7.0	11.4 ± 4.7	.339
Cr (µmol/L, mean ± SD)	73.3 ± 15.8	70.1 ± 13.1	.353
TG (mmol/L, mean ± SD)	1.4 ± 1.0	1.4 ± 0.7	.747
CHOL (mmol/L, mean ± SD)	4.0 ± 0.9	3.8 ± 0.9	.519
HDL-C (mmol/L, mean ± SD)	1.1 ± 0.3	1.0 ± 0.3	.375
LDL-C (mmol/L, mean ± SD)	2.5 ± 0.8	2.3 ± 0.7	.073

D3, Dolutegravir (DTG) plus lamivudine (3TC). TLE, Tenofovir (TDF) plus Lamivudine (3TC) and Efavirenz (EFV).

ALT = alanine aminotransferase, AST = aspartate aminotransferase, BMI = body mass index, CHOL = total cholesterol, Cr = creatinine, HDL-C = high density lipoprotein cholesterol, HVL = high viral load, LDL-C = low density lipoprotein cholesterol, LVL = low viral load, OIs = opportunistic infections, TBIL = total bilirubin, TG = triglyceride.

* *P* < .05.

### 3.2. Comparison of virological suppression

**Figure 1.**
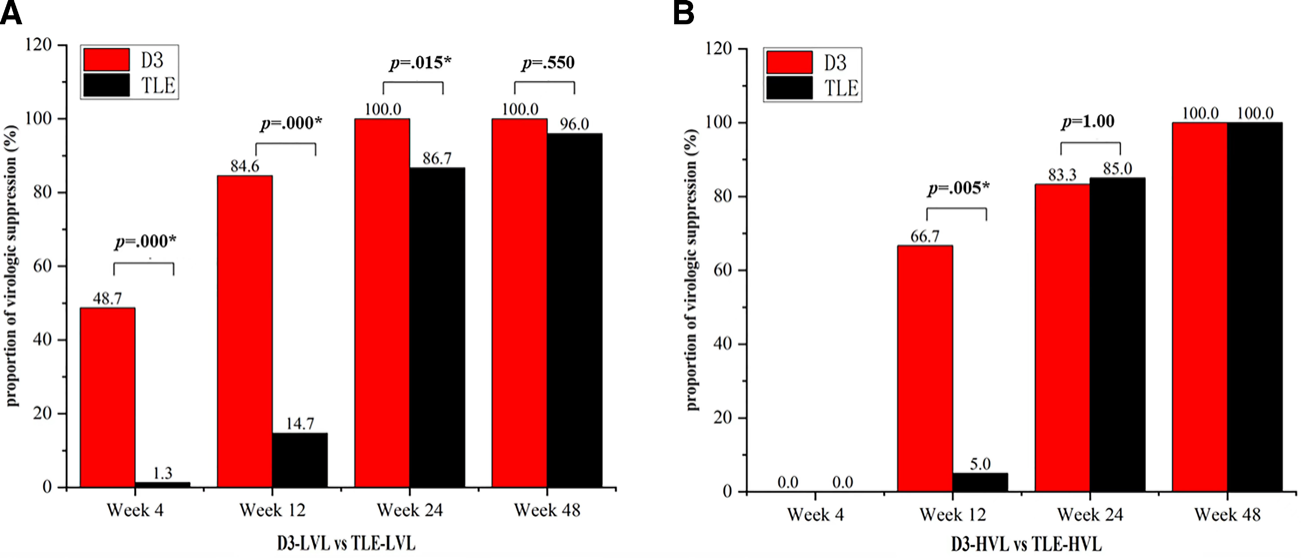
Comparison of virologic suppression in subgroups. (A) Proportion of HIV RNA < 50 copies/mL in D3-LVL versus TLE-LVL. (B) Proportion of HIV RNA < 50 copies/mL in D3-HVL versus TLE-HVL. D3, dolutegravir (DTG) plus lamivudine (3TC). TLE, tenofovir (TDF) plus lamivudine (3TC) and efavirenz (EFV). HIV = human immunodeficiency virus, HVL = high viral load, LVL = low viral load, RNA = ribonucleic acid. *P < .05.

When HIV-1 RNA ≤ 500,000 copies/mL at baseline (LVL subgroups), the proportion of participants with HIV RNA < 50 copies/mL at week 4, 12, 24 was 8.7% (19/39), 84.6% (33/39), 100% (39/39) in D3 group, higher than 1.3% (1/75), 14.7% (11/75), 86.7% (65/75) in TLE group (*P* < .05) (Fig. 1A). All participants achieved virologic suppression in D3-LVL subgroup at week 48, compared with 96.0% (72/75) in TLE-LVL subgroup (*P* > .05). No virologic rebound occurred in D3-LVL subgroup at week 48, while 3 participants in TLE-LVL subgroup found recurrent HIV-1 RNA range from 51 to 66 copies/mL.

When HIV-1 RNA > 500,000 copies/mL at baseline (HVL subgroups), no participants in both groups achieved virological suppression at week 4 and all did at week 48 without virologic rebound (Fig. 1B). 66.7% (4/6) was observed with HIV RNA < 50 copies/mL in D3-HVL subgroup at week 12 versus 5.0% (1/20) in TLE-HVL subgroup (*P* < .05). The proportion was similar at week 36 with 83.3% (5/6) and 85.0% (17/20) respectively in 2 subgroups (*P* > .05).

### 3.3. Comparison of immunological response

**Figure 2.**
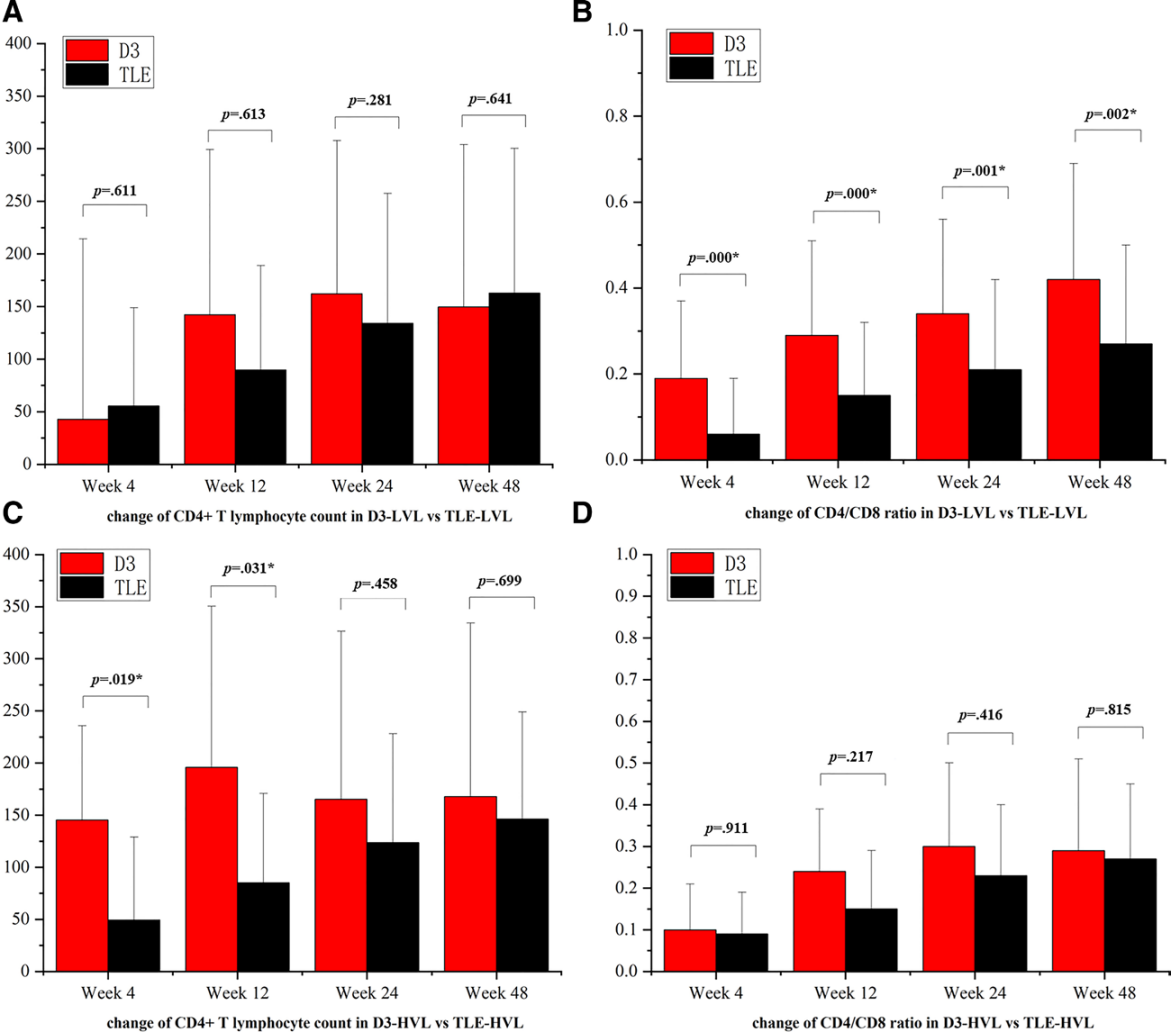
Comparison of immunological response in subgroups. (A) Change of CD4^+^ T lymphocyte count in D3-LVL versus TLE-LVL. (B) Change of CD4/CD8 ratio in D3-LVL versus TLE-LVL. (C) Change of CD4^+^ T lymphocyte count in D3-HVL versus TLE-HVL. (D) Change of CD4/CD8 ratio in D3-HVL versus TLE-HVL. D3, dolutegravir (DTG) plus lamivudine (3TC). TLE, tenofovir (TDF) plus Llamivudine (3TC) and efavirenz (EFV). LVL = low viral load. HVL = high viral load. *P < .05.

CD4^+^ cell count and CD4/CD8 ratio increased in both groups following ART initiation. Mean changes of CD4/CD8 ratio from baseline to week 4, 12, 24, 48 were 0.06, 0.15, 0.21, 0.28 in D3-LVL subgroup, higher than in TLE-LVL subgroup with 0.07, 0.15, 0.23, 0.26 (*P* < .05) (Fig. 2B). Elevated level of CD4^+^ cell count was similar in D3-LVL and TLE-LVL subgroups (*P* > .05) (Fig. 2A).

Mean changes of CD4^+^ cell count from baseline to week 4, 12, 24, 48 were 145.3, 195.8, 165.2, 167.8 in D3-HVL subgroup compared with 49.5, 85.1, 123.6, 146.2 in TLE-HVL subgroup, with *P* < .05 at week 4 and 12 (Fig. 2C). Mean changes of CD4/CD8 ratio were similar in D3-HVL and TLE-HVL subgroups (*P* > .05) (Fig. 2D).

### 3.4. Comparison of drug adverse effect

**Figure 3.**
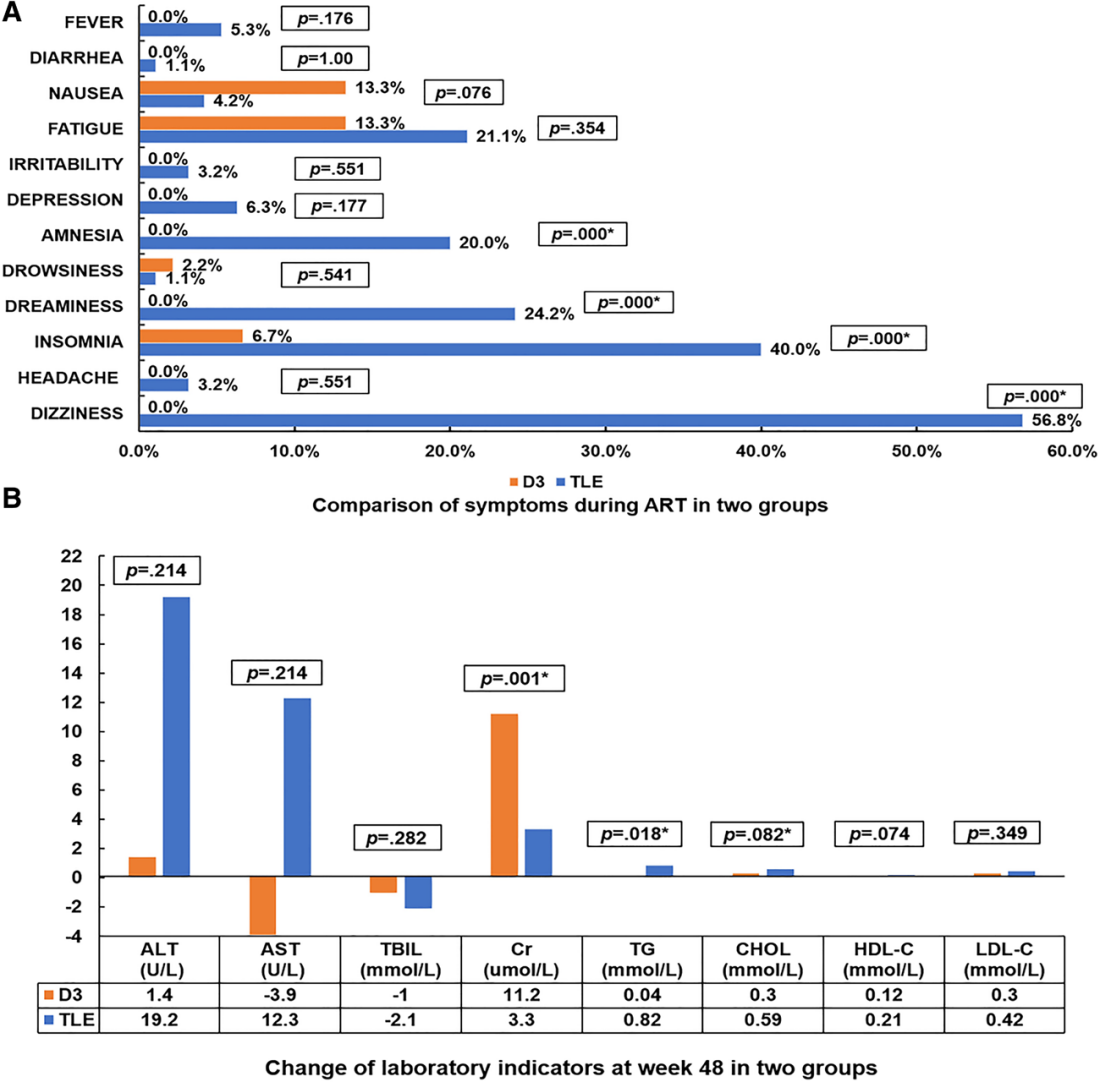
Comparison of drug adverse effect in D3 and TLE groups. (A) Comparison of symptoms during ART in two groups; (B) Change of laboratory indicators at week 48 in two groups. D3, dolutegravir (DTG) plus lamivudine (3TC). TLE, tenofovir (TDF) plus lamivudine (3TC) and efavirenz (EFV). ALT = Alanine aminotransferase, AST = aspartate aminotransferase, CHOL = total cholesterol, Cr = creatinine, HDL-C = high density lipoprotein cholesterol, LDL-C = low density lipoprotein cholesterol, TBIL = total bilirubin, TG = triglyceride. *P < .05.

More complaint of dizziness, insomnia, dreaminess and amnesia was documented in TLE group (*P* < .05) (Fig. 3A). No severe adverse event occurred in both groups. Elevated level of creatinine from baseline to week 48 was higher in D3 group and elevated level of triglyceride was higher in TLE group (*P* < .05) (Fig. 3B). No significant difference was found between changes of alanine aminotransferase, aspartate aminotransferase, bilirubin, total cholesterol, high density lipoprotein cholesterol and low density lipoprotein cholesterol in 2 groups.

### 3.5. HIV-1 DNA response in D3 group

**Figure 4.**
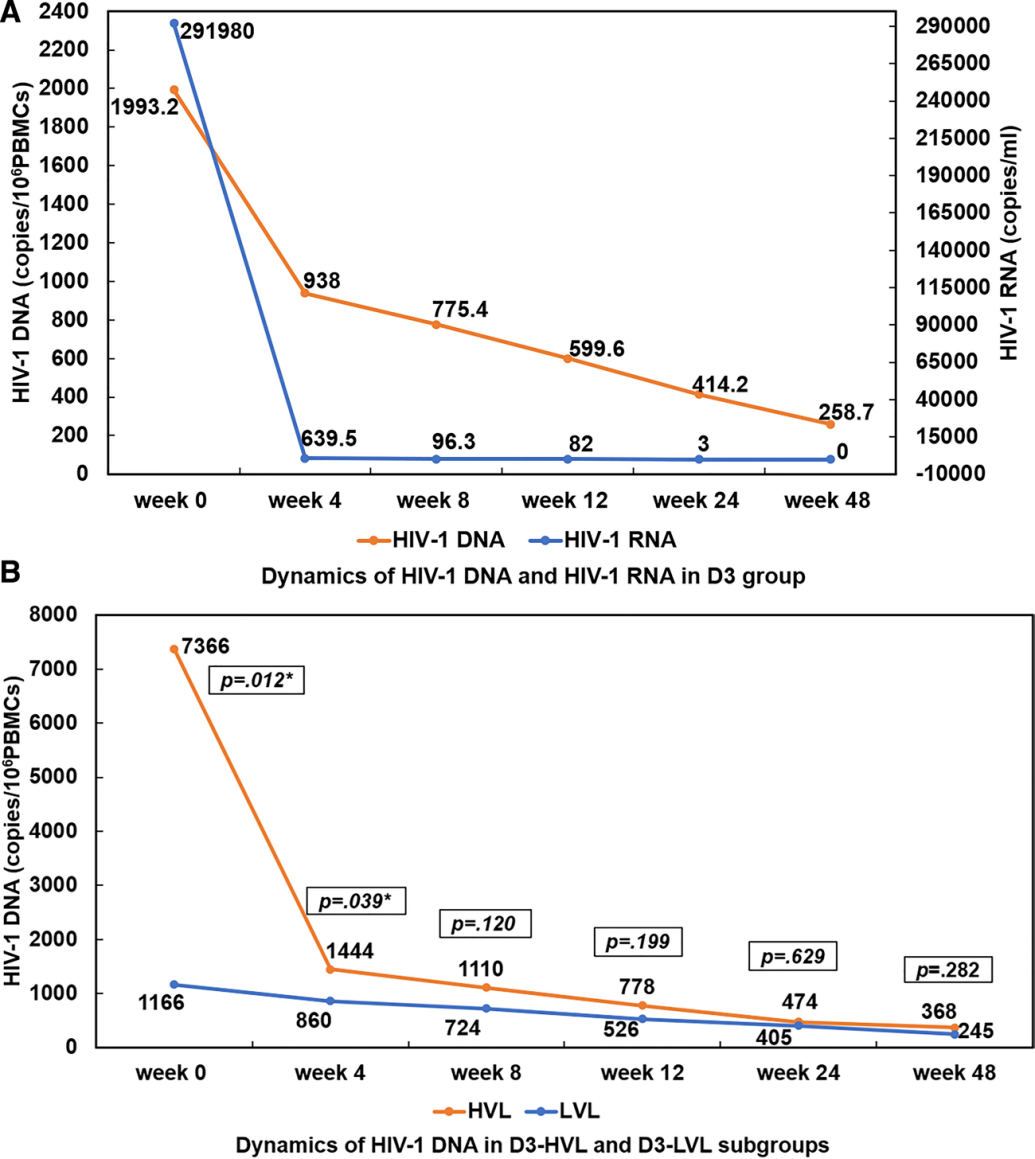
HIV-1 DNA Response in D3 Group. (A) Dynamics of HIV-1 DNA and HIV-1 RNA in D3 group; (B) Dynamics of HIV-1 DNA in D3-HVL and D3-LVL subgroups. D3, dolutegravir (DTG) plus lamivudine (3TC). DNA = deoxyribonucleic acid, HIV = human immunodeficiency virus, HVL = high viral load, LVL = low viral load, RNA = ribonucleic acid. *P < .05.

Significant correlation between HIV-1 DNA and HIV-1 RNA was observed at baseline with Pearson correlation coefficient of 0.376 (*P* = .011) (Fig. 4A). HIV-1 DNA decreased along with HIV-1 RNA in both subgroups after DTF + 3TC initiation. HIV-1 DNA level was significantly lower in D3-LVL than D3-HVL subgroup at baseline and week 4 (*P* < .05), but then no obvious difference was observed at week 8, 12, 24 and 48 (*P* > .05) (Fig. 4B).

## 4. Discussion

DTG + 3TC has been one of the most common recommended dual ART regimens due to the advantage of its potent antiviral effectiveness with high genetic barrier to resistance, rare severe side effects, and few drug-drug interactions.^[6,7]^ PADDLE study revealed 48-week virologic suppression rate of 95% of INSTI/lamivudine dual therapy in ART-naive patients with HIV-1 RNA ≤ 100,000 copies/mL.^[8]^ The GEMINI-1 and GEMINI-2 phase III trials demonstrated the 96-week and 144-week noninferior virologic efficacy of DTG + 3TC in ART-naive HIV-1-infected individuals versus DTG plus TDF/emtricitabine with the proportion of achieving HIV-1 RNA < 50 copies/mL over 85% in both groups.^[9,10]^ Similar results were reported in studies including some real-world data about 2-drug regimen (2DR) of DTG + 3TC supporting dolutegravir-based 2DRs as good therapeutic options for HIV-1 infection, either ART-naïve or virologically suppressed on previous antiretroviral regimen.^[16–26]^ In China, DTG + 3TC was recently recommended as a first-line regimen for ART-naïve individuals in 2021.^[2]^ Its real-world data for efficacy and safety was limited but have attracted extensive attention. In this ongoing prospective real-world study for ARV-naive HIV-1-infected individuals, we observed that DTG + 3TC presented more rapid and potent virological suppression than TDF + 3TC + EFV at week 4, 12, 24 in LVL subgroups and at week 12 in HVL subgroups (*P* < .05). At week 48, all participants achieved HIV-1 RNA < 50 copies/mL in DTG + 3TC group versus 96.8% (92/95) in TDF + 3TC + EFV group without significant difference. The result furtherly confirmed the antiviral potence of DTG + 3TC in ART-naïve HIV-1 infected adults in China across baseline viral load strata, especially in the early stage following ART initiation. Meanwhile the antiviral efficacy was more stable since no virologic rebound was found at week 48 in DTG + 3TC group.

Immunological response was usually evaluated by CD4^+^ cell count and CD4/CD8 ratio. In a recent test-and-treat study, median change from baseline to week 24 in CD4^+^ cell count and CD4/CD8 ratio under DTG/3TC treatment was 170 cells/mL and 0.26 respectively.^[26]^ Another study reported that 12-week and 24-week CD4^+^ cell count increased more rapidly in the 2DR arm than 3DR arm.^[24]^ In our study, immunological response as CD4^+^ cell count and CD4/CD8 ratio increase occurred in both DTG + 3TC and TDF + 3TC + EFV groups. Mean change of CD4/CD8 ratio were higher in D3-LVL than TLE-LVL subgroup at each scheduled visit (*P* < .05), while CD4^+^ cell count increased significantly in D3-HVL than TLE-HVL subgroup at week 4 and 12 (*P* < .05). Better immunological efficacy was revealed in DTG + 3TC group no matter what level of the baseline HIV RNA.

DTG is a second-generated INSTI with rare severe side effects and few drug-drug interactions^[6,7]^ and 3TC is a potent NRTI with well-proven safety profile. Thus, DTG + 3TC is considered as a more convenient and safer dual regimen compared with those including TDF, NNTRIs or boosted protease inhibitors. Consistent with the theory and previous reports, we observed less complaint of dizziness, insomnia, dreaminess and amnesia and lower elevated level of triglyceride in DTG + 3TC group verse TDF + 3TC + EFV group. The symptoms and hypertriglyceridemia are commonly caused by efavirenz. Meanwhile significantly higher elevated level of creatinine was observed in DTG + 3TC group, due to the inhibition of creatinine tubular secretion by DTG.^[27,28]^

To date total HIV DNA is the most widely used and studied marker for HIV reservoir, notwithstanding it includes integrated and nonintegrated viral genomes without differentiating the defective forms from the latent ones that can produce infectious viruses.^[12]^ In this real-world study, we observed positive correlation between HIV-1 DNA and HIV-1 RNA at baseline, and both of them decreased during ART with DTG + 3TC. HIV-1 DNA decayed to a non-significantly lower level since week 8 in DTG + 3TC subgroups, suggesting the potent inhibiting ability on total HIV-1 DNA of DTG + 3TC regimen in individuals with high baseline HIV-1 RNA.

There were some limitations in this study. First, the sample size of totally 45 participants in DTC + 3TC group is quite small, though it is more than other real-world studies found before. Second, we analyzed the data from baseline to just 48 weeks after ART initiation. A larger sample size and a longer follow-up are needed in this ongoing prospective study to document the durability of DTG + 3TC regimen in terms of efficacy and safety. Finally, regularity of HIV-1 DNA on DTG + 3TC would be better analyzed with a control group.

## 5. Conclusion

DTG + 3TC achieved virological suppression more rapidly and stably versus TDF + 3TC + EFV in ART-naïve HIV-1-infected adults in this real-world study across baseline viral load strata, with better immunological response and less adverse drug effect of symptoms and hypertriglyceridemia. Meanwhile it could reduce total HIV-1 DNA effectively. Thus, DTG + 3TC is a potent regimen for ART-naïve individuals with HIV-1 infection.

## Author contributions

**Conceptualization:** Jin Li, Dabiao Chen.

**Data curation:** Huijun Zhong, Yanhao Wang.

**Formal analysis:** Dabiao Chen, Zhanlian Huang.

**Investigation:** Zhiwei Wen, Yanzhang Du.

**Supervision:** Jin Li, Sichun Yin.

**Writing – original draft:** Dabiao Chen.

**Writing – review & editing:** Jin Li.
